# Cortical modulation of thalamic function during cortical spreading depression- Unraveling a new central mechanism involved in migraine aura"

**DOI:** 10.1186/1129-2377-14-S1-I6

**Published:** 2013-02-21

**Authors:** Anna P Andreou, Till Sprenger, Peter J Goadsby

**Affiliations:** 1 Headache Group- Department of Neurology, University of California, San Francisco, San Francisco, CA, USA; 2 Anesthetics, Pain Medicine and Intensive Care Section, Division of Medicine, Imperial College London, London, UK; 3 Department of Neurology and Division of Neuroradiology, University Hospital Basel, Basel, Switzerland

## 

The thalamus is a key structure in migraine pathophysiology[1]. Direct cortico-thalamic connections provide important interactions on both cortical and thalamic structures[2]. Cortical spreading depression (CSD), believed to underlie the pathophysiology of migraine aura[3], would be expected to influence sensory responses of thalamic neurons, through such corticothalamic interactions.

To investigate this, a CSD was induced while recording neuronal activity from ipsilateral thalamic neurons responding to electrical stimulation of dural vessels. CSD induced a transient increase of spontaneous activity for 30-150s. Following this activity, in 43% of the studied neurons, spontaneous neuronal activity, as well as, Aδ- and C-fiber activity in response to dural vessel stimulation, was significantly enhanced for 15-90min by 94±17%, 27±6% and 109±33%, respectively. In 38% of neurons, spontaneous neuronal firing, Aδ- and C-fiber activity were significantly decreased following CSD by a maximum of 44±3%. Interestingly, none of the short or long-lasting effects of a single CSD within the thalamus were altered following trigeminal ablation. In a different experimental group, multiple waves of K^+^-induced CSDs significantly inhibited neuronal activity, compared to a single CSD. Thalamic recordings during a single CSD were further compared to CSD-evoked responses in the ipsilateral and contralateral trigeminocervical complex (TCC). CSD induced both inhibitory and excitatory responses on ipsilateral and contralateral second order neurons, through different mechanisms of action, as previously described[4]^,5^. In comparison, CSD induced a higher degree of neuronal activation within the ipsilateral sensory thalamus, compared to the facilitatory evoked-activity of CSD within the TCC (ipsilateral spontaneous activity:94±17% *vs* 27±11%; C-fiber activity:109±33% *vs* 36±5%).

The data demonstrate that CSD markedly alters neuronal firing of ipsilateral third order thalamic neurons, independent of peripheral trigeminal inputs. This provides a new mechanism by which CSD may indeed induce central head pain via cortico-thalamic circuits and may shed more light on the relationship between aura and headache.
